# Embryological etiology of pancreaticobiliary system predicted from pancreaticobiliary maljunction with incomplete pancreatic divisum: a case report

**DOI:** 10.1186/s12893-018-0385-4

**Published:** 2018-08-02

**Authors:** Yukihiro Sanada, Yasunaru Sakuma, Naohiro Sata

**Affiliations:** 1 Department of Surgery, Jichi Medical University, 3311-1 Yakushiji, Shimotsuke City, Tochigi 329-0498 Japan; 2 Department of Transplant Surgery, Jichi Medical University, Shimotsuke, Japan

**Keywords:** Pancreaticobiliary maljunction, Incomplete pancreatic divisum, Embryology, Ventral pancreas, Endoscopic retrograde cholangiopancreatography

## Abstract

**Background:**

The genesis of the “complex type” classification of pancreaticobiliary maljunction (PBM) is unclear, and the pancreaticobiliary anatomy is also varied according to each case. We encountered a patient with PBM and incomplete pancreatic divisum (PD). We herein discussed about the embryological etiology of pancreaticobiliary system predicted from PBM with incomplete PD.

**Case presentation:**

A 67-year-old man was found to have a dilatation of the common bile duct (CBD) during a medical examination at 62 years of age. The dilatation of the CBD subsequently progressed, and he was admitted to our hospital for surgical treatment. Magnetic resonance cholangiopancreatography revealed a dilatation from the common hepatic duct to the middle bile duct with PBM. Endoscopic retrograde cholangiopancreatography from the papilla of Vater revealed the pancreatic main duct via the pancreatic branch duct, and PBM with dilatation of the CBD and incomplete PD were revealed. We performed an extrahepatic bile duct resection and hepaticojejunostomy because of high risk of malignant transformation. Taping and transection of the bile duct without dilatation on the pancreatic side were performed, and thereafter, two orifices of the common channel and ventral pancreatic duct were ligated. The level of amylase in the bile was 7217 IU/L, and a histological examination of the CBD showed an inflammatory change of CBD, not a malignant transformation.

**Conclusion:**

It is somewhat easy to identify the pancreatobiliary anatomy when the cause of embryology of both PBM and PD is thought to be an abnormal embryology of the ventral pancreas.

## Background

The “complex type” classification of pancreaticobiliary maljunction (PBM) is rare, [1] and in particular, PBM with pancreatic divisum (PD) is classified a “complex type” of PBM. The genesis of the “complex type” classification of PBM is unclear, and the pancreaticobiliary anatomy is also varied according to each case. Therefore, it is occasionally difficult to understand the anatomy and pathophysiology of patients with PBM and PD. In addition, the misunderstanding of pancreaticobiliary anatomy affects a decision of the transection line during biliary diversion procedure.

We encountered a patient with PBM and incomplete PD. We herein discussed about the embryological etiology of pancreaticobiliary system predicted from PBM with incomplete PD in order to understand the pancreaticobiliary anatomy.

## Case presentation

**Fig. 1:**
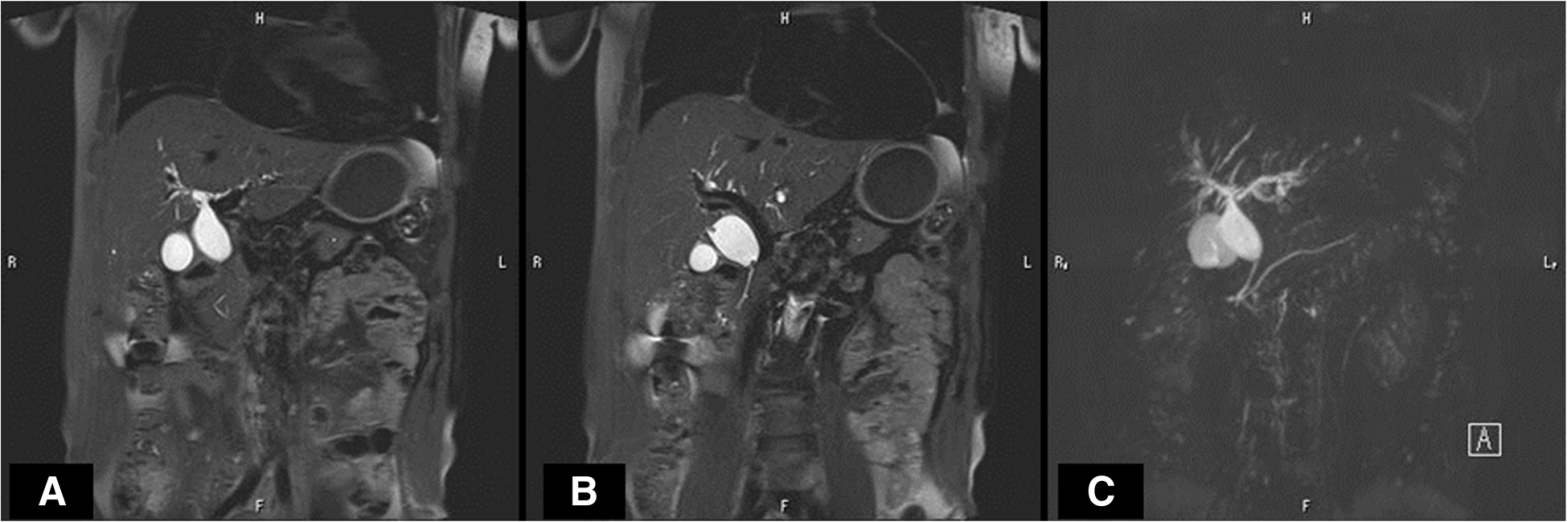
Magnetic resonance cholangiopancreatography (MRCP). MRCP revealed a dilatation from the common hepatic duct to the middle bile duct (**a** and **b**) with pancreaticobiliary maljunction (**c**)

**Fig. 2:**
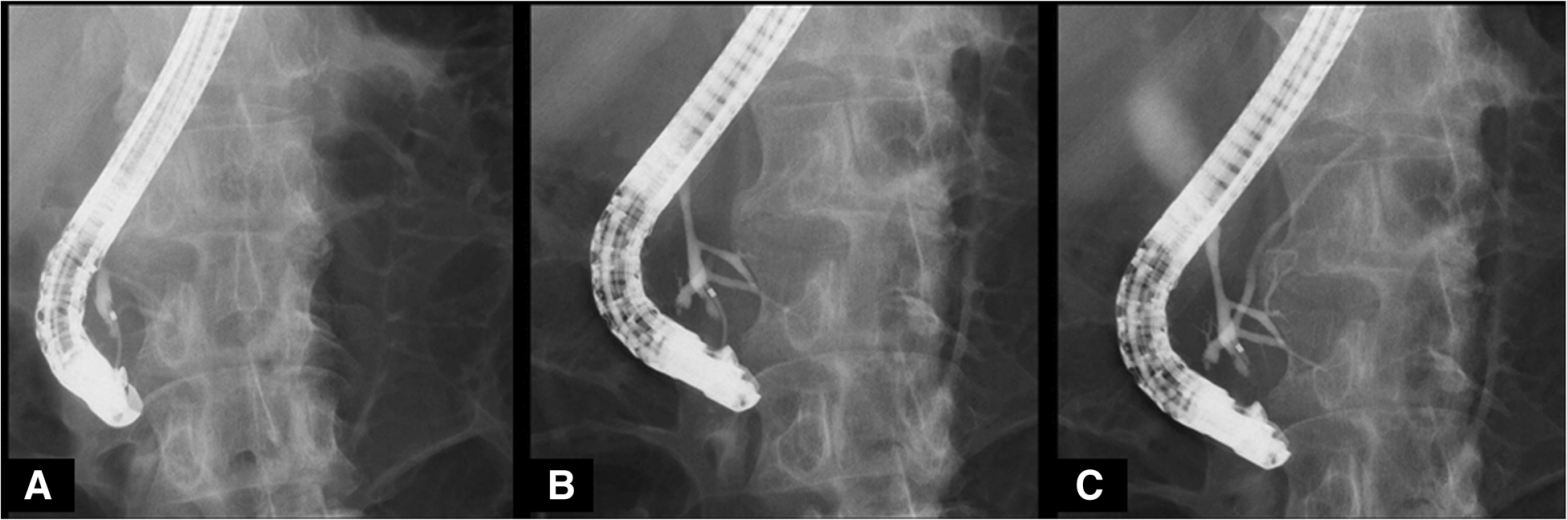
Endoscopic retrograde cholangiopancreatography (ERCP). ERCP from the papilla of Vater revealed the pancreatic main duct via the pancreatic branch duct (**a** and **b**). Pancreaticobiliary maljunction with dilatation of the common bile duct (26 mm) and incomplete pancreas divisum were revealed (**c**)

**Fig. 3:**
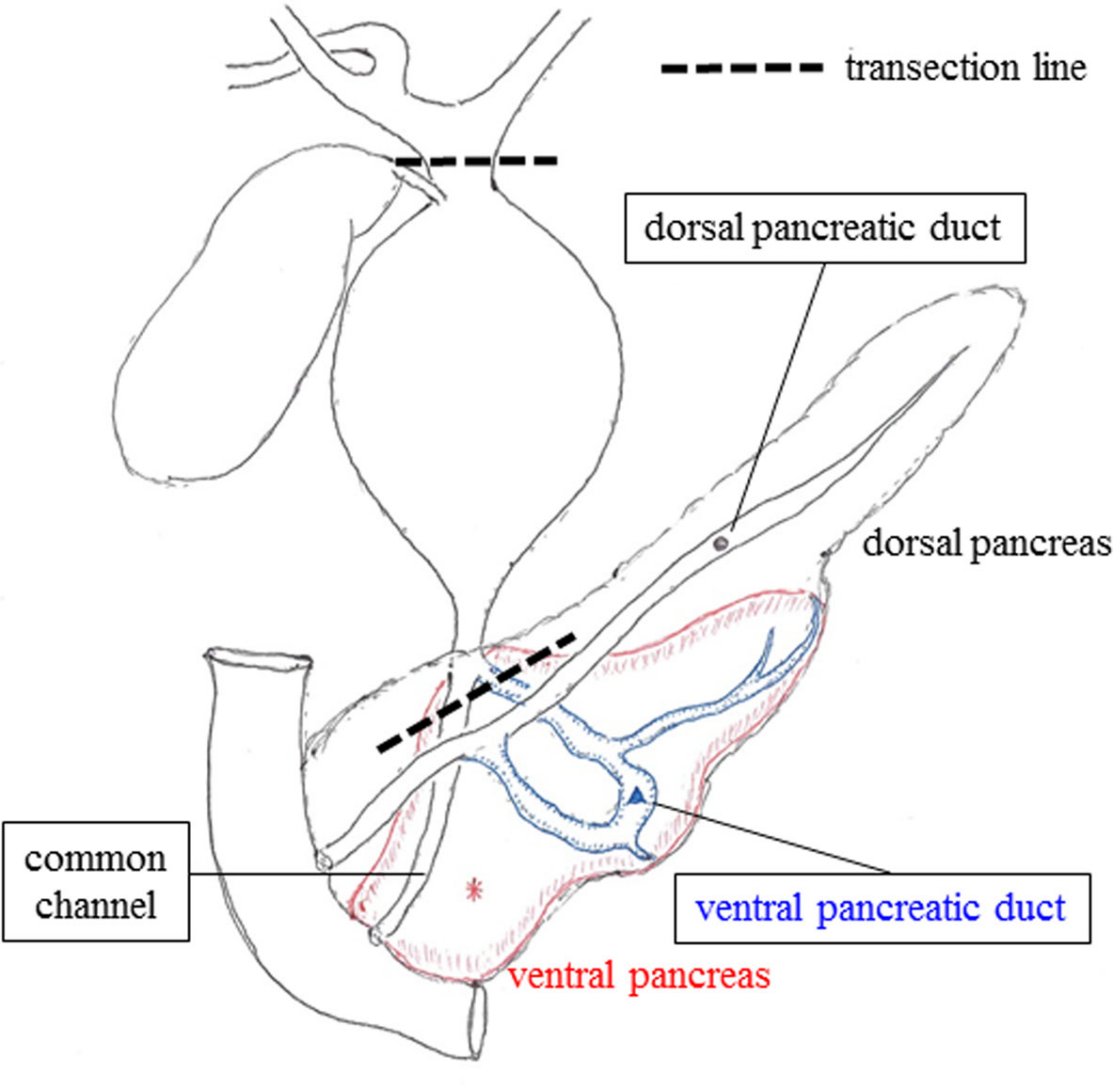
Anatomical schema of the pancreaticobiliary system in this patient. This patient was diagnosed with the “complex type” classification of pancreaticobiliary maljunction with incomplete pancreas divisum. Extrahepatic bile duct resection and hepaticojejunostomy were performed based on the transection line of this schema

A 67-year-old man was found to have a dilatation of the common bile duct (CBD) (19 mm) during a medical examination at 62 years of age. The dilatation of the CBD subsequently progressed (26 mm), and he was admitted to our hospital for surgical treatment. Abdominal computed tomography revealed a dilatation of the CBD with no tumor or stone. Magnetic resonance cholangiopancreatography revealed a dilatation from the common hepatic duct (CHD) to the middle bile duct (Fig. 1a and b) with PBM (Fig. 1c). Endoscopic retrograde cholangiopancreatography (ERCP) from the papilla of Vater revealed the pancreatic main duct via the pancreatic branch duct (Fig. 2a and b). PBM with dilatation of the CBD (26 mm) and incomplete PD were revealed (Fig. 2c). Figure 3 shows a schema of this case with dilatation of the CBD and PBM, and incomplete PD in which the ventral pancreatic duct joined the dorsal pancreatic branch duct was observed.

We planned an extrahepatic bile duct resection and hepaticojejunostomy because of high risk of malignant transformation. Laparotomy was performed by a right hypochondrium incision. Taping and transection of the bile duct without dilatation on the pancreatic side were performed, and thereafter, two orifices of the common channel and ventral pancreatic duct were ligated (Fig. 3). The transection line of the CHD without dilatation was identified using cholangiography (Fig. 3), and then, the CBD was resected. The level of amylase in the bile was 7217 IU/L, and a histological examination of the CBD showed an inflammatory change of CBD, not a malignant transformation.

The postoperative course was good and uneventful, and the patient was discharged from the hospital on postoperative day 9. The patient is doing well at 1.5 years after surgery.

## Discussion

The “complex type” classification of PBM is rare, [1] and in particular, PBM with PD is considered a “complex type” classification of PBM. The embryological mechanism of PBM is unclear; however, there are strong arguments for an abnormal embryology of the ventral pancreas until embryonic week four [2, 3]. In contrast, it is conceivable that the embryological mechanism of PD occurs by an abnormal fusion of the ventral and dorsal pancreatic ducts because the ventral pancreas fuses with the dorsal pancreas at embryonic weeks 6–7 [4]. That is, PBM and PD are congenital anomalies that develop in the embryo at an early stage, and they may be the result of bile and pancreatic duct misarrangement. However, the genesis of the “complex type” classification of PBM is unclear, and the anatomy is also varied according to each case. In addition, few patients have been reported with PBM and PD [5]. Therefore, it is occasionally difficult for physicians and surgeons to understand the anatomy and pathophysiology of patients with PBM and PD. We herein encountered a patient with PBM and incomplete PD, and we considered that PBM and incomplete PD occurred in this case due to abnormal embryology of the ventral pancreas. If the embryological etiology of PBM and PD is an abnormal embryology of the ventral pancreas, the genesis and anatomy of the “complex type” classification of PBM is easy to understand. This case supports the idea that the embryology of PBM and PD is based on abnormal embryology of the ventral pancreas.

Although the recommended treatment for a biliary dilatation with the “complex type” classification of PBM is an extrahepatic bile duct resection and hepaticojejunostomy (biliary diversion procedure), [3] selecting the transection lines of the CBD is occasionally difficult. It is important to leave as little as possible of the bile duct unresected, and in this case, the bile duct on the pancreatic side was transected at the bifurcation of the common channel and ventral pancreatic duct so as not to leave the bile duct because drainage from the ventral pancreatic duct to the dorsal pancreatic duct can be identified by ERCP. The patient is doing well without having an acute pancreatitis due to insufficient drainage of the ventral pancreatic duct. Therefore, it is important for patients with the “complex type” classification of PBM to clear the dynamics of the ventral pancreatic juice by preoperative ERCP, and it is possible to undergo a complete resection of the bile duct on the pancreatic side.

## Conclusion

It is difficult to identify the “complex type” pancreatobiliary anatomy of PBM; however, it is somewhat easy to identify when the cause of embryology of both PBM and PD is thought to be an abnormal embryology of the ventral pancreas.

## Abbreviations

CBD: Common bile duct
CHD: Common hepatic duct
ERCP: Endoscopic retrograde cholangiopancreatography
PBM: Pancreaticobiliary maljunction
PD: Pancreas divisum
